# Health timeline: an insight-based study of a timeline visualization of clinical data

**DOI:** 10.1186/s12911-019-0885-x

**Published:** 2019-08-22

**Authors:** Andres Ledesma, Niranjan Bidargaddi, Jörg Strobel, Geoffrey Schrader, Hannu Nieminen, Ilkka Korhonen, Miikka Ermes

**Affiliations:** 1 Tampere University, Faculty of Medicine and Health Technology, Tampere, Finland; 2 College of Medicine & Public Health, Flinders University, Medical Sciences Rd, Adelaide, Australia; 3 Discipline of Psychiatry, The Queen Elizabeth Hospital, South Australia, University of Adelaide, 28 Woodville Rd, Woodville South, Adelaide, Australia; 4 Country Health Local Health Network, South Australia, and Flinders University, Medical Sciences Rd, Adelaide, Australia; 5 VTT Technical Research Center Ltd, Tekniikankatu 1, Tampere, Finland

**Keywords:** Clinical data, Data visualization, Health informatics, Electronic health record, Insight-based methodology

## Abstract

**Background:**

The increasing complexity and volume of clinical data poses a challenge in the decision-making process. Data visualizations can assist in this process by speeding up the time required to analyze and understand clinical data. Even though empirical experiments show that visualizations facilitate clinical data understanding, a consistent method to assess their effectiveness is still missing.

**Methods:**

The insight-based methodology determines the quality of insights a user acquires from the visualization. Insights receive a value from one to five points based on a domain-specific criteria. Five professional psychiatrists took part in the study using real de-identified clinical data spanning 4 years of medical history.

**Results:**

A total of 50 assessments were transcribed and analyzed. Comparing a total of 558 insights using Health Timeline and 576 without, the mean value using the Timeline (1.7) was higher than without (1.26; p<0.01), similarly the cumulative value with the Timeline (11.87) was higher than without (10.96: p<0.01). The average time required to formulate the first insight with the Timeline was higher (13.16 s) than without (7 s; p<0.01). Seven insights achieved the highest possible value using Health Timeline while none were obtained without it.

**Conclusions:**

The Health Timeline effectively improved understanding of clinical data and helped participants recognize complex patterns from the data. By applying the insight-based methodology, the effectiveness of the Health Timeline was quantified, documented and demonstrated. As an outcome of this exercise, we propose the use of such methodologies to measure the effectiveness of visualizations that assist the clinical decision-making process.

**Electronic supplementary material:**

The online version of this article (10.1186/s12911-019-0885-x) contains supplementary material, which is available to authorized users.

## Background

Researchers estimate that clinical data will grow [1], from 153 exabytes in 2013 to 2314 in 2020 [2]. Electronic health records (EHRs) constitute most of the clinical data. These records are the “purest type” of electronic clinical data obtained at the point of care [3]. EHRs collected over time represent a patient’s clinical history. Healthcare professionals rely on them for diagnosis and treatment.

As clinical data increases in volume, so does the potential value for healthcare providers to benefit from it. IT-based information systems can summarize and visualize clinical data. Health Informatics is the “interdisciplinary study of the design, development, adoption and application of IT-based innovations in healthcare services delivery” [4]. Numerous research efforts have addressed the need to understand clinical data by designing and building a variety of visualizations [5, 6].

North suggests that the purpose of visualizations is to help readers derive insights [7]. A measure of an effective visualization is its ability to generate new insights that go beyond predefined data analysis tasks [8]. The effectiveness of clinical data visualizations is an area of active research [5, 6, 9]. Graphical representation techniques have been addressed in several studies [10]. One significant example was reported by Goetz who demonstrated that data presentation greatly affects the understanding of a person’s health [11]. Thus, visualization concepts applied to clinical data should enable healthcare professionals to obtain insights about the condition of the patient more comprehensively and efficiently than traditional textual or tabular presentations.

Researchers have implemented several visualization tools for clinical data [5]. A literature survey identified fourteen computerized tools for visualizing EHRs [6]. The articles describe in detail the design and implementation of these tools. However, they do not use a structured method, such as the insight-based methodology, to perform assessments.

We extracted data from the Australian “My Health Record” initiative [12] and developed a computerized solution to visualize clinical data chronologically. The solution was named Health Timeline to emphasize the significance of time in the clinical data. The software organizes and displays the clinical data in an interactive timeline using visual enhancements to facilitate readability. This article documents the assessment of Health Timeline which applies the insight-based methodology. Five psychiatrists took part in the study by reviewing the clinical data of five de-identified patients. The psychiatrists were instructed to use the “thinking aloud process”. This process consists of verbalizing the thought pattern followed while inspecting the data. Their findings were recorded using a voice recording software. These findings were later transcribed and analyzed following the insight-based methodology.

### Related work

The findings of Lesselroth and Pieczkiewicz [5], as well as those of Rind and colleagues [6] are further discussed. The study reported in this article relates to other computerized solutions. The key distinction is the assessment methodology.

#### Scope

The focus of this study is on time-based visualizations of clinical data (EHR) and the assessment methodology used to validate them. Time-based visualizations are graphical representations of data collected over time. The research literature has a large number of data visualization techniques that vary in their strategies. However, we refined our search to include only visualization tools based on time, and the longitudinal nature of the data. The search was narrowed down further to only include those techniques that were used in the context of clinical data. We were particularly interested in the assessment methodology used to evaluate these visualizations.

#### Review of similar solutions in the healthcare context

LifeLines is a computerized tool that displays clinical data [13] using dots positioned along horizontal lines [14]. A study showed that participants responded 50% faster to a “post-experimental memory test” (*p*<0.004) [15]. LifeLines was extended in a second version with support for aggregation of temporal events [16]. The focus was on emphasizing prevalence and temporal order of the clinical data. A study revealed that the clinicians were able to confirm hypotheses on the hospital length of stay of patients. LifeLines [13] and LifeLines2 [16] are Java software applications, thus they must be installed in a Java-capable device. These tools provide data filters to narrow down the data exploration and enable the user to focus on certain aspects of the timeline.

TimeLine is a visualization software that displays EHRs chronologically [17]. The data is grouped by categories and displayed along a visual timeline. No assessment of the software was reported in the article. Timeline [17] is the closest application to Health Timeline. It features web support, EHR interfacing and a timeline representation of data with a focus on oncology. Timeline also has a large number of features such as causal models, imaging files, data search, disease progression visualization and data category toggling. It is probable that using Timeline involves a learning curve as it offers several features that would require the user to become acquainted with.

LifeFlow is a visualization tool that summarizes data in sequences using temporal spacing of events [18]. One physician took part in a briefing interview about the visualization of patient transportation data. EventFlow is a drug prescription pattern visualization [19]. A study on the use of asthma drugs was conducted to identify patterns that complied with regulations. LifeFlow and EventFlow are also software applications that require installation. The data are not visualized in a timeline but instead the representation is chronologically ordered as a series of events and outcomes. These visualizations are optimized for understanding the causes and outcomes of patient admissions to hospital.

#### Assessment methods

Bertini and colleagues [20] made a strong case for the objective assessment of visualization tools. A literature review on assessment methods for information visualization reports a number of practical cases and proposes a classification of these methods. The Visual Data Analysis and Reasoning (VDAR) classification group is relevant to our study because it emphasizes the decision-making process, knowledge discovery and visual data analysis. We found that no assessments of this kind have been conducted using clinical data and medical experts.

#### Summary

Time-based visualizations have been found helpful in several use cases. However, without a systematic assessment method, it is difficult to demonstrate how they improve the understanding of the data. To provide a contribution towards the good practice of assessment methods for clinical data visualizations, we conducted and documented the assessment of the Health Timeline using the insight-based methodology. This methodology has been previously used in bioinformatics [8, 21–23] and well-being data analysis [9, 24].

### Key contribution

The key contribution is to test out the insight-based methodology for the assessment of a clinical data visualization of EHRs. To our understanding, researchers have not documented an insight-based evaluation in this specific context.

#### Evaluating visual data analysis and reasoning

The VDAR classification group proposed by Lam and colleagues [25] is relevant to this study due to the nature of the clinical data. The purpose of our study is to measure the degree to which a time-based visualization can assist the data analysis and the reasoning process of a clinician when analyzing the clinical history of a patient. In the context of clinical data, we found in the research literature that studies tend to focus on the usability of the interactions with the visualization system. Other studies conduct briefing interviews and questionnaires to try to assess the participants’ understanding of the data. Nevertheless, a consistent evaluation methodology is still missing.

The purpose of our study is to measure the degree to which a time-based visualization can assist the data analysis and reasoning process of a clinician when analyzing the clinical history of a patient. In the context of clinical data, we found in the research literature that studies tend to focus on the usability of the interactions with the visualization system. Other studies conduct briefing interviews and questionnaires to try to assess the participants’ understanding of the data. Nevertheless, a consistent evaluation methodology is still missing.

#### Contextualizing the insight-based methodology

The insight-based methodology provides an assessment method focusing on the insights generated by a visualization [7]. In this approach, the insights formulated by the visualization audience are assessed in a Likert scale from 1 to 5. To contextualize the insight-based methodology to the clinical data domain, we consulted with two subject matter experts to assist us in establishing the assessment criteria of the insights.

#### From bioinformatics to clinical data

The insights gained in this study are analogous to those observed in the bioinformatics context because they demonstrate different degrees of completeness and accuracy in the understanding of the data represented by the visualization. In the bioinformatics context, predetermined information deemed as correct and complete, was used to compare the insights obtained in the study [8, 21, 22]. The same is true for our study, since two professional psychiatrists reviewed the evaluation criteria. Due to their experience, these psychiatrists were qualified to evaluate the degree of completeness and accuracy of the insights. These experts were also acquainted with the clinical data used in our study.

The insight-based methodology could be used in future studies to quantify the effectiveness of clinical data visualizations. This study provides details on the application of the methodology, including the process required to establish criteria to assess the insight value. The insight value is a key component to analyze and interpret the results of the evaluation. We hope to provide enough documentation about the steps followed to apply the insight-based methodology. The purpose is to provide a case study on evaluating visualizations using the insight-based methodology contextualized to the healthcare domain.

## Methods

In this study we documented the application of the insight-based methodology. This process involved the recording of insights, the formulation of a consistent criteria for insight assessment and the application of those criteria to collect and analyze the results. To this extent, we define the goals of the study as follows:

1. To apply the insight-based methodology to assess the Health Timeline visualization using clinical data and Healthcare professionals.
2. To document the assessment process so that it may serve as a reference for future studies on clinical data visualizations.

In this section we present the experiment protocol and the hypothesis that we proposed on the outcome of the study. We also present the Health Timeline and explain its design rationale. The counterpart of the Health Timeline is a table showing the clinical history of the patient in textual format. We refer to this table as the “baseline”. This section also provides information about the clinical data used in the study, as well as the order in which the visualizations were displayed to the participants. Finally, we explain the metrics used to analyze and interpret the results of the study in accordance with the insight-based methodology.

### Hypothesis

We propose the following hypothesis: a time-based visualization will enable clinicians to obtain valuable insights regarding the clinical condition of patients. A valuable insight demonstrates a complete and accurate understanding of the data. This understanding of the data is corroborated by the evaluation criteria used to assess the insight value. The criteria were designed and reviewed by subject matter experts. Valuable insights will translate to better patient diagnosis. This in turn will result in better decision-making processes thereby improving patient outcomes.

### Health timeline

The Health Timeline was designed to provide a simple and interactive way to review a patient’s medical history using a time-based visualization. For healthcare professionals, the visualization software aims to assist in the decision-making process when assessing the overall status of the patient.

The Health Timeline is a cloud-based software that acquires data from the Medicare and Pharmaceutical Benefits Scheme (PBS) claims systems as part of Australia’s “My Health Record” initiative. These claims include a multitude of information as well as pharmaceutical aspects of patient data, including consultations, laboratory tests, in-patient admissions and other relevant information. The application handles the consent, sign up and compliance processes for using the clinical data. It has received conformance certification from Australia’s National E-Health Transition Authority (NEHTA) and the Commonwealth Department of Health for use with production servers and deployment in clinical practice.

#### Graphical representation and interactivity

**Fig. 1:**
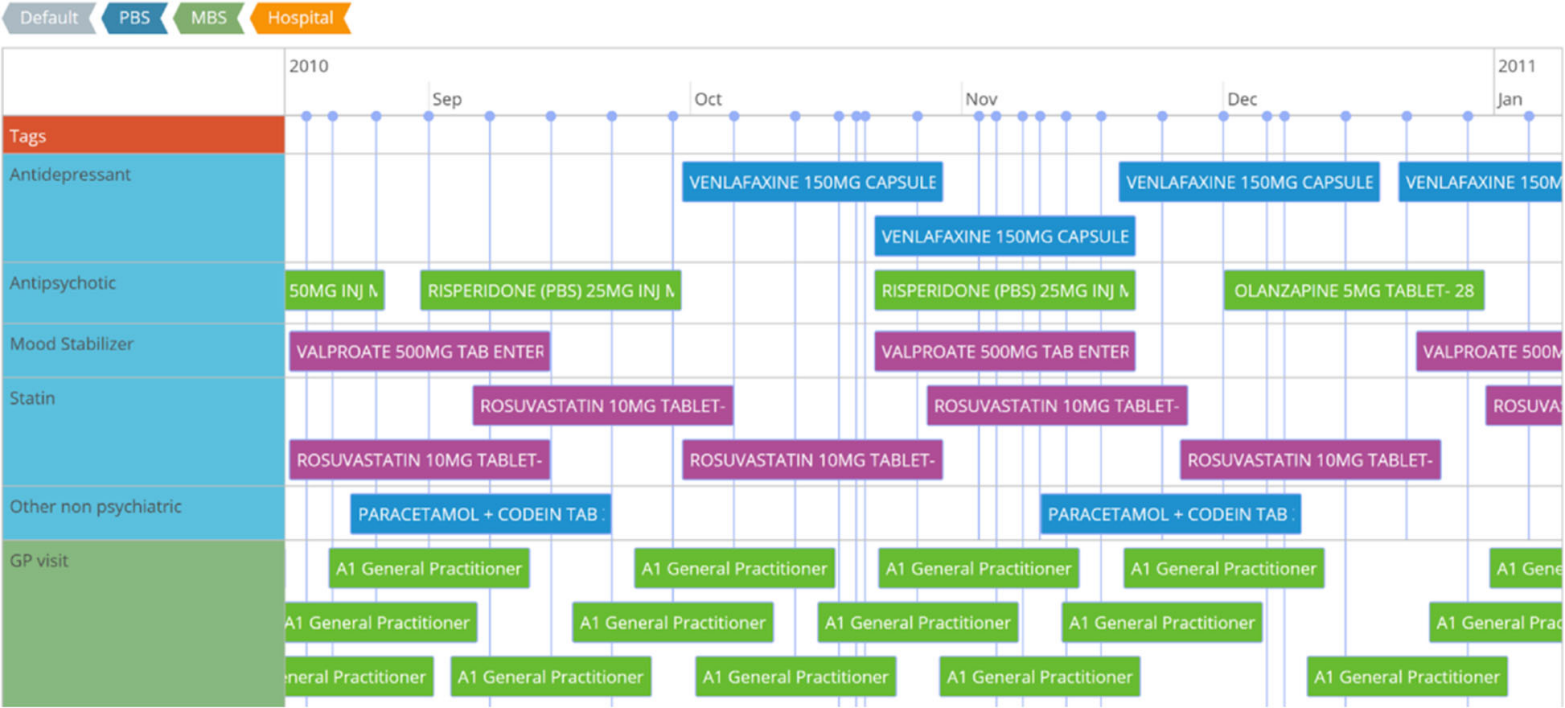
Health Timeline. Timeline visualization showing the collection of EHRs of a patient used in the study

The events in the timeline are grouped in adjacent rows. Each event is depicted as a box. The boxes are grouped in rows with labels on the left edge of the timeline. The events are shown at the corresponding start date position displayed on the horizontal axis. The width of the event box is adjusted to the corresponding end date. The group labels correspond to domains that are clinically relevant, for example medical treatments, visits to the general practitioner, psychiatric consultations and laboratory tests. Users can interact with the timeline by adjusting the displayed time frame (start and end dates). This is done by zooming in and out or panning left and right. Figure 1 shows an example of the Health Timeline portraying the clinical history of a patient.

#### Design rationale and requirements

The rationale behind the design of Health Timeline is to provide a web-based (usable with any device with a web browser) visualization tool for clinicians to assist in the process of understanding the overall condition of a patient. The Health Timeline extracts information from EHR systems and presents the data in an interactive interface.

The design principles followed to develop Health Timeline were based on a simple and comprehensive visualization. In the “Related work” section, we identified similar visualization tools. These tools supersede the Health Timeline in terms of features and complexity. However, in this study, the participants did not receive an introduction or explanation on how Health Timeline software works, this was part of the intended strategy of the study. That is, to design and develop a simple yet comprehensive visualization that would require no introduction to clinicians. In other words, we aim to provide an intuitive visualization of clinical data that clinicians can understand without requiring training.

The Health Timeline builds on similar time-based visualizations and tries to address the challenge of representing data clearly and in a meaningful way without overwhelming the user with excessive details and complex interfaces. During the first assessment, the participants became acquainted with the Health Timeline visualization. In contrast, more complex visualization software, such as LifeFlow [18] or EventFlow [19] would probably require the participants to get acquainted with the tool before conducting the experiment.

The key requirements of Health Timeline came from Australia’s “My Health Record” initiative and were gathered from interviews with clinicians and Information Technology experts with a background in data visualization. These requirements are summarized as follows:

- Web-based visualization software
- Acquisition of data from Medicare and PBS
- Cloud-hosted application software
- A clean and simple user-interface. For the purpose of the study, no tutorials or explanations were given to the participants. This was done to “stress test” the design of the graphical interface.
- Interactivity for data exploration (time adjustment via panning and zooming)

### Data representation baseline

**Fig. 2:**
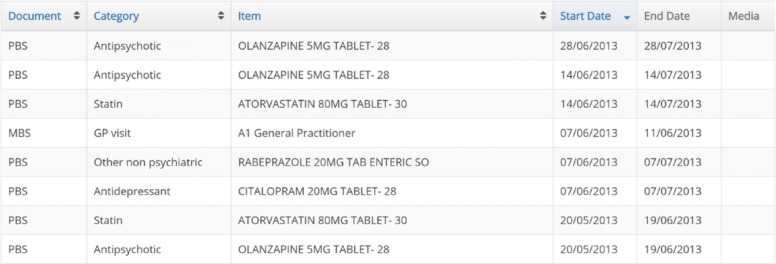
Visualization Baseline. The baseline visualization showing a set of EHRs of a patient used in the study

The baseline representation of the clinical data is the current interface that clinicians have to the EHRs. The tabular data show in the baseline is the starting point of the study and contains the same information as the Health Timeline. The tabular data representation allows the user to sort the events chronologically, alphabetically, by document type and category. Figure 2 shows an example the tabular format representation of the baseline.

### Insight-based methodology

The study documented in this article follows the insight-based methodology as proposed by North [7]. An insight is defined as “the capacity to gain an accurate and deep understanding” (Oxford, 2016). As presented in the literature survey, the most prevalent approach to evaluate clinical data visualization is to conduct briefing interviews or to measure the performance of the user conducting a set of predefined tasks. By contrast, the insight-based methodology focuses on recognition and quantification of insights gained from exploratory use of the data visualization. In the context of this study, an insight is a unit of discovery based on observation [8, 9, 21, 22, 24].

Insights have a quantifiable value based on the assessment criteria. The proposed criteria take into consideration the following characteristics of an insight [7, 8, 21–23]:

- **Observation**: The observation or finding provided by the participant during the process of analyzing the data via a representation.
- **Time**: The amount of time taken to reach the insight.
- **Domain Value**: The value, importance, or significance of the insight.
- **Hypotheses**: Some insights enable users to identify a new relevant hypothesis.
- **Directed versus Unexpected**: Directed insights are those that answer specific questions. Unexpected insights are those that were not considered in the design of the study.
- **Correctness**: Insights can be correct or incorrect depending on the data represented in the visualization. Some insights are incorrect conclusions that result from misinterpreting the data visualization. For our study, the insights formulated by the participants need to be clinically valid assessments on the patient’s condition.

Time, domain value, hypothesis and correctness are characteristics included in this study. We did not compile a set predefined of insights that the participants were required to formulate. Instead, the focus of the study was on allowing the participants to explore the data and formulate observations freely. This was designed to simulate the typical use case in which a healthcare provider is presented with a patient history and has to become acquainted with the data in a short span of time before the consultation. Therefore, the directed versus unexpected characteristic was not used in our study.

#### Domain value criteria and insight value coding

**General Criteria:** The domain value of an insight is determined using a five-point Likert scale as suggested in the methodology [7] and applied in previous studies [8, 9, 21–23]. The value depends on the insight’s complexity and depth. An insight is complex if it can associate more than one element in the data by providing a relationship or association between them, for example; “if “a” increases then “b” decreases”. The depth is the degree to which the underlying reasons behind the data are explained, that is, a plausible explanation was given that could justify the state of the data. For instance, an explanation for values outside of the recommended range for certain physiological measurements could indicate the presence of a disease. In our study, we recognized that additional refinement was needed for the evaluation criteria used to assess the insight value, due to the complexity of the data.

**Longitudinal Nature:** Previous studies focused on the visualization of “static” data. The data used for this study was essentially chronological since it was comprised of clinical histories of psychiatric patients. The medications, appointments, inpatient and outpatient treatments were all visualized over time for the duration of the clinical history. Therefore, suitable criteria should evaluate the insights considering the temporal nature of the data.

**Criteria Formulation and Review Process:** To determine the insight value, we applied the complexity and depth criteria as used in previous studies. However, as the Insight-based Methodology had not been previously applied in this context and lacked a precedent, to overcome this limitation, we asked two professional psychiatrists to assess the usefulness of the insights using a five-point Likert scale.

The consulting psychiatrists analyzed a sample of 100 insights (extracted from the assessments made by the participants) and evaluated them according to what they considered informative and helpful in understanding the patient’s condition. The consulting psychiatrists were acquainted with the data and thus were able to understand the underlying condition and overall health of the patients. The insights provided to the consulting psychiatrists included the patient identifier number and the text transcribed manually from the voice recording. This allowed them to have a reference to the original data to objectively determine if an insight was accurate and meaningful in understanding the medical history and clinical condition of the patient.

**Agreed Criteria and Value Coding:** The result of this process was a set of rules that enabled us to systematically and consistently assign a numeric value (domain value) to the insights. For example, an insight with a value of one, describes an event in the timeline without associations to other events or without possible explanations for the causes behind the event. Examples of these insights are: “the patient has an elevated heart-rate” or “the patient is taking medication for diabetes”.

Insights that describe associations of events regarding their frequency, pattern and irregularities were evaluated with two points. These insights connect multiple occurrences of similar events and show that the clinician can track down a pattern. For example, insights like “the patient has regular appointments with a General Practitioner” or “the patient is taking a high dose of the medication with regular frequency”. These insights have a time component as the participant identifies a regularity or irregularity in their occurrence.

The insights that include theories that could explain some or all the symptoms and thus the reasoning behind a prescription, or a specialist appointment, were evaluated with three, four or five points depending on how much information they could combine to produce a valid clinical statement. These insights could be considered a hypothesis and could explain valid clinical scenarios that result in drug treatment, laboratory tests, specialists’ visits and in-patient treatments. The following insight provides an illustration: “the patient has experienced depression and anxiety, that would explain the prescription and regular use of the drug treatment and also visits to the specialists, this also ties together an emergency episode in January 2013 and another admission in June, overall the patient’s mental health improved towards the summer and it seems that changing the drug treatment improved the outlook”. Table 1 summarized the criteria for each value with examples extracted from the transcription of the assessments.

**Table 1:** Criteria used to determine the insight value

Value	Criteria	Example
1	Describe the data. No patterns or periodicity spotted. Values described as “low” or “high”. No awareness of the times an event appears in the dataset.	“this is a patient on injectable antipsychotic medications”
2	Describe periodicity or frequency of an event. Found patterns, irregularities and amount of repetitions. No conjectures or assumptions.	“the patient has quite a number of GP visits in 2011, 2012 and 2013”
3	Requires a conjecture or assumption. Try to explain why an event or value is repeating, missing, following a pattern. Speculation on the treatment, status, follow-up, treatment or behavior of the patient. Prediction on the future status of the patient based on a single event. No correlation with other events. Single conjecture explaining one phenomenon	“patient also has an investigation suggesting that he has some metabolic disease”
4	Conjecture or assumption about two or more events. Explanation of probable cause and effect. Ties two events or phenomena together with a probable reason or explanation. Not all the elements in the dataset are explained some relationships remain unknown to this insight.	“in summary I think it is a patient with psychotic illness”
5	Hypothesis that ties together the discovered elements and events into one possible explanation. Explains relationship between events. Explains probable underlying reasons of the events. Ties together all the events mentioned beforehand.	“overall this patient presents quite of a complex picture of mainly depression probably complicated with psychotic component anxiety”

Examples are provided from the insights we obtained during the study

#### Thinking aloud process

The insight-based methodology recommends two mechanisms to record the insights, the “thinking aloud process” and the use of a written diary to record the steps taken during the data analysis. We aimed at making the participation in the study as simple as possible since the participants were professional psychiatrists with busy schedules. For this reason, we opted to use the “thinking aloud process” and capture the insights, comments and other statements via phone calls and recording software. We later transcribed the recordings manually to proceed to the analysis stage of the study.

The “thinking aloud process” is one of the most common techniques in usability studies [26]. It consists of asking the participant to verbally express the thought process while using the system under testing. In this study, we asked the participants to follow a link received via email and once they were ready to begin, they called a number and started the “thinking aloud process” of verbalizing their thoughts as they explored the data. This was an effective and convenient way to conduct the study since the participants had the freedom to conduct the assessments at their convenience.

By using the “thinking aloud process” we were able to capture the actions the participants were conducting during the assessments, for example scrolling, panning and zooming to a particular time frame.

### Clinical data

De-identified patient data used for the purpose of the study was obtained with consent from mental health patients with various psychiatric disorders [27]. The patients were either outpatients seen in clinics in rural South Australia (77) or patients reviewed in an urban hospital emergency department (63). Ethical approval for the study was obtained from the South Australian Health Ethics Research Ethics Committee and the Medicare Australia Ethics Committee. Each patient gave written consent to use their data for research purposes. From the pool of 144 patients, the de-identified data of five patients was selected for this evaluation based on the complexity of diagnosis and treatment.

#### Patient data selection

The patient selection was based on the complexity of the diagnosis. All five cases had a diagnosed mental disorder and received continuous care and monitoring throughout their treatment. Three cases had in-patient episodes. A fourth patient was compliant with the treatment and the disorder was managed throughout the recorded period. A fifth patient had substantial changes in overall mental health.

#### Clinical data included in the study

The selected data included Pharmaceutical Benefit claims, Medicare Benefits claims and hospitalization dates over 3 years between 2012 and 2014. The data also included visits to General Practitioners, specialists, laboratory tests, emergency hospital admissions, drug treatment, among other categories. The claims records were sourced from Medicare Australia and hospitalization events were sourced from SA Health. Australia’s Pharmaceutical Benefits Scheme (PBS) contains information about the type of medication, amount of medication supplied, and the date of supply, while the Medicare Benefits Scheme (MBS) contains information on tests and services by Medicare eligible practitioners and the associated date of the service. The PBS and MBS measures were relabeled into clinical terminologies by psychiatrists involved in the project. The data used are considered a clinical history of a patient as part of the EHR.

### Experiment protocol

The participating psychiatrists received written instructions on how to conduct the assessments. The instructions contained 10 web links used to access a web portal that displayed the patient data. Five assessments were conducted with the Health Timeline and another five with the tabular format. The web links displayed one patient data set at a time in either representations.

A log-out timer was set after accessing each link. This was done to provide the psychiatrist with three minutes to analyze the available information and formulate insights. Subsequently, the psychiatrist was automatically logged out of the system and the assessment session was concluded. The psychiatrists accessed the links with their personal computers and thus they were familiar with the browser and operating system of their choice. The psychiatrist’s observations during the assessment sessions were recorded on an answering machine over a phone connection.

As in previous studies [8, 9, 21–23], the assessments were transcribed from the recorded sessions. The transcriptions were annotated with the times at which the insights were mentioned by the participant. The insights were evaluated using the previously specified criteria.

#### Visualization presentation and order

All five patients were presented with both visualizations to account for control. The display order was controlled to first show the Health Timeline and direct any bias towards this visualization upon starting the assessments. There was no introduction or instructions given to the participants on how the Timeline works. The idea was to build a visualization that would be intuitive from the start.

The participants conducted the same assessments independently. The participants were asked to make one assessment at a time. The order was the same for all the participants. Each assessment featured the clinical data of a single patient. The patient clinical data was presented to the participant twice, one time in tabular data and another in the Health Timeline visualization. The participants conducted a total of 10 assessments, 5 with the Health Timeline and another 5 with the tabular data. The visualizations were alternated throughout the study. The first assessment used the Health Timeline, the second tabular data, the third Health Timeline, the fourth tabular data and so on. The order of the patients (clinical data) was also controlled so that no consecutive visualization would show the same patient data.

#### Time constraints

The experiment was intended to be performed by clinicians. As such, time was a major constraint. Empirically we have observed that taking no more than 30 min of their time would be ideal. Keeping each assessment to no more than three minutes was considered to be short enough for them to be willing to take part in the experiment. The 30-min time frame was determined by the three-minute duration of each assessment, a total of 10 assessments which showed the clinical data of five patients with both visualizations, Health Timeline and the textual data. Additionally, we allowed clinicians to take the assessments at their convenience, so if a clinician was able to spare three minutes, then that would be enough to conduct one of the 10 assessments.

#### Sample size

A total of 10 psychiatrists were invited to take part in the study. Five of them accepted the invitation. Each participant conducted a total of 10 assessments, 5 with the Health Timeline and 5 in textual data. The presentation order was the same for all participants.

#### Participants

We recruited five professional psychiatrists to participate in the assessments. The participants had experience in the field and had previously worked with similar patients as those used in the study. The participants also expressed their written consent to participate.

#### Data analysis

A total of 121 min of audio were transcribed. The transcribed text for each insight was organized using a spreadsheet that included the patient identifier, the participant identifier and the time at which the insight was mentioned. The data set produced was saved as a comma separated value file for further analysis and contained the following attributes (Additional file 1):

- The **experimentId** refers to the sequential identifier number of the assessments.
- The **patientId** refers to an identifier created for the sole purpose of this study and is used to link together the assessments with the de-identified patient data. The identifier does not contain clinical or personal information.
- The **assessmentId** refers to the assessment during which the participants used either the Timeline or the baseline to conduct their observations.
- The **visualization** refers to either the Health Timeline (referred to as timeline) or the baseline (referred to as table).
- The **time** refers to the time at which the insight began to be voiced by the participant. In some cases, multiple insights share the same starting time indicating that they were given by the participant during the same sentence (thought process).
- The **insight** refers to the textual insight as transcribed from the audio.
- The **value** refers to the domain value of the insight which follows the criteria detailed in this article.

#### Metrics

As in previous studies [8, 9, 21–23], we analyzed the data and applied the following metrics to evaluate the data representations:

- **Metrics per assessment**: For each assessment we calculated the average number of insights, as well as their average and cumulative value.
- **Number of insights**: the total count of insights across all the assessments. We also counted the number of insights separated by their value (from one to five points).
- **Time to first insight**: the time required by the participant to formulate the first insight regardless of its value. Additionally, we also calculated separately the insight time for each of the values.

#### Statistical tests

Mann–Whitney–Wilcoxon (MWW) was used to calculate the statistical significance of the metrics per assessment and the time to first insight since they are not normal distributions. MWW is the recommended method for these distributions [28, 29].

The statistical significance of the total number of insights was calculated using standard Chi squared because the two populations could be treated as categorical data. The insight value corresponds to the categories, the values are discrete from 1 to 5 using a Likert scale. Therefore, the statistical significance can be obtained with Chi squared [29].

## Results

The participants completed all 50 assessments per the protocol. The total duration of resulting recordings was 78,783 s, mean and standard deviation was calculated for the distributions (69.47*μ*±51.63*σ*). The recordings resulted in 1,134 transcribed insights (per participant 22.68*μ*±9.70*σ*).

### Metrics per assessment

**Fig. 3:**
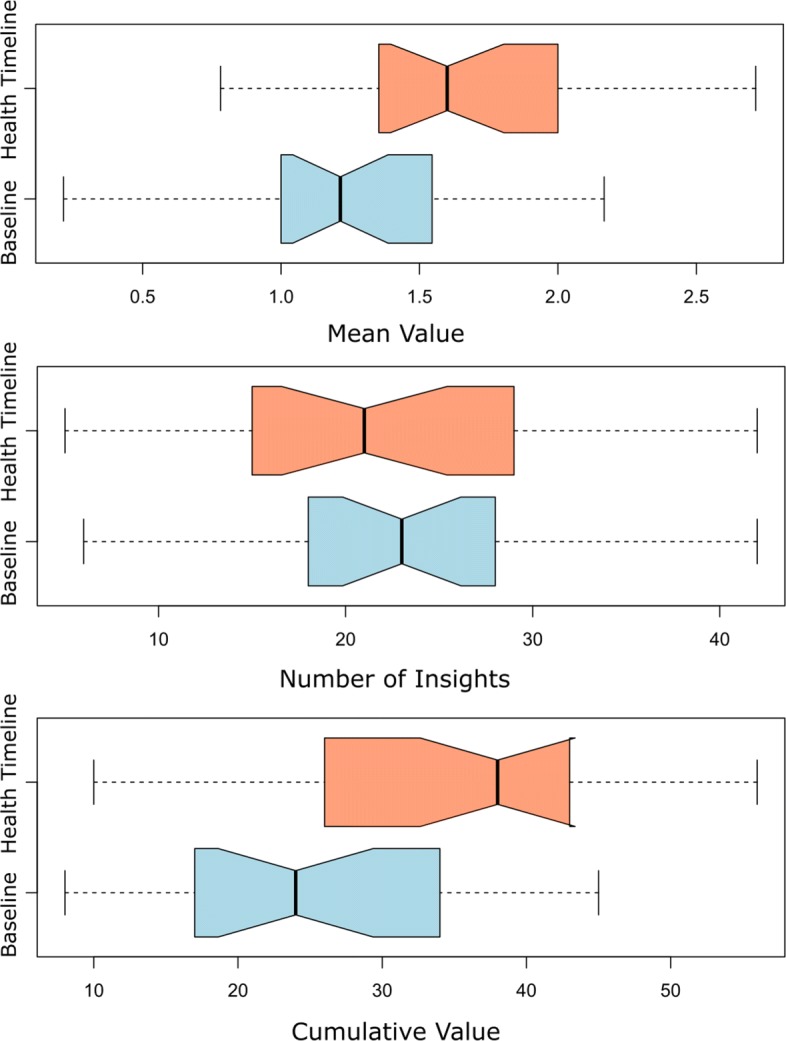
Metrics per assessment. The box plots represent the number of insights, mean and cumulative value of the insights per assessment

The collection of insights was analyzed to calculate the average number of occurrences, the mean and cumulative value for each assessment. Figure 3 illustrates, using box plots, the difference in the metrics per assessment and Table 2 summarizes the characteristics of the distributions such as the minimum, maximum, mean, median and standard deviation.

**Table 2:** Metrics per Assessment

Metric	Baseline	Health Timeline	*p*-values
Number of insights	*min*=6	*min*=5	0.70
	*max*=42	*max*=42	
	*μ*=23.04	*μ*=22.32	
	*Md*=23	*Md*=21	
	*σ*=±10.43	*σ*=±9.12	
Cumultive value	*min*=8	*min*=10	0.01
	*max*=45	*max*=56	
	*μ*=24.96	*μ*=34.68	
	*Md*=24	*Md*=38	
	*σ*=±10.96	*σ*=±11.87	
Mean value	*min*=0.21	*min*=0.78	0.01
	*max*=2.17	*max*=2.71	
	*μ*=1.26	*μ*=1.70	
	*Md*=1.21	*Md*=1.60	
	*σ*=±0.52	*σ*=±0.57	

The table shows the insights generated by participants using Health Timeline and the visualization baseline per assessment. Statistical significance is shown in the *p* column using Mann-Whitney U tests

The distribution of the number of insights per assessment was not statistically significant, with the Timeline having an average of 22.32 insights compared to the baseline with 23.04 (*p*=0.7047). The mean value with the Timeline (1.70) was higher than the baseline (1.26; *p*=0.01). Similarly, the cumulative value with the Timeline (34.68) was significantly higher than the baseline (24.96; *p*=0.01).

### Number of insights

The value of each insight was collected from the dataset. The distribution is shown in Table 3 as a total count of insights by value. The total distribution of insights was not statistically significant. The Timeline (558) had a lower count that the baseline (576; *p*=0.7). Table 4 shows the characteristics of the value distribution. The average and median insight value with the Timeline (*μ*=1.0833; *Md*=1; *σ*=±1.10) was higher than the baseline (*μ*=1.55; *Md*=2; *σ*=±1.25; p <0.01)

**Table 3:** Insight Distribution

Metric by Insight Value	Baseline	Health Timeline	Ratio	*p*-values
Any	576	558	1.16	0.70
1	175	114	0.65	0.03
2	98	178	1.82	0.01
3	71	78	1.10	0.53
4	10	32	3.20	0.01
5	0	7	—	Non-significant

The table shows the distribution of the insights according to their domain value. The ratio column shows the observations of the Health Timeline divided by the baseline. The *p*-values are obtained using Wilcoxon Signed-Rank Test

**Table 4:** The table compares the distributions showing the minimum, maximum, median, average and standard deviation of the two populations, in this case these are the Health Timeline and the baseline representation

Metric	Baseline	Health Timeline	*p*-values
Insight Value	*min*=0	*min*=0	<0.01
	*max*=4	*max*=5	
	*μ*=1.0833	*μ*=1.55	
	*Md*=1	*Md*=2	
	*σ*=±1.10	*σ*=±1.25	

The statistical tests were conducted with Chi-squared since the populations can be treated as categorical data (value 1 to 5 each comprise their own category)

Table 3 shows that the distribution was statistically significant for insights of value 1 (*p*=0.03), 2 (*p*<0.01), 4 (*p*<0.01) and 5 (*p*<0.01) but not for value 3 (*p*=0.53). Insights of value 3 or more demonstrate an understanding of the data that can detect patterns. A total of 81 insights were observed using the baseline compared to 118 with the Health Timeline. Additionally, only 7 insights of value five were generated and all occurred with the Timeline.

### Time to first insight

**Fig. 4:**
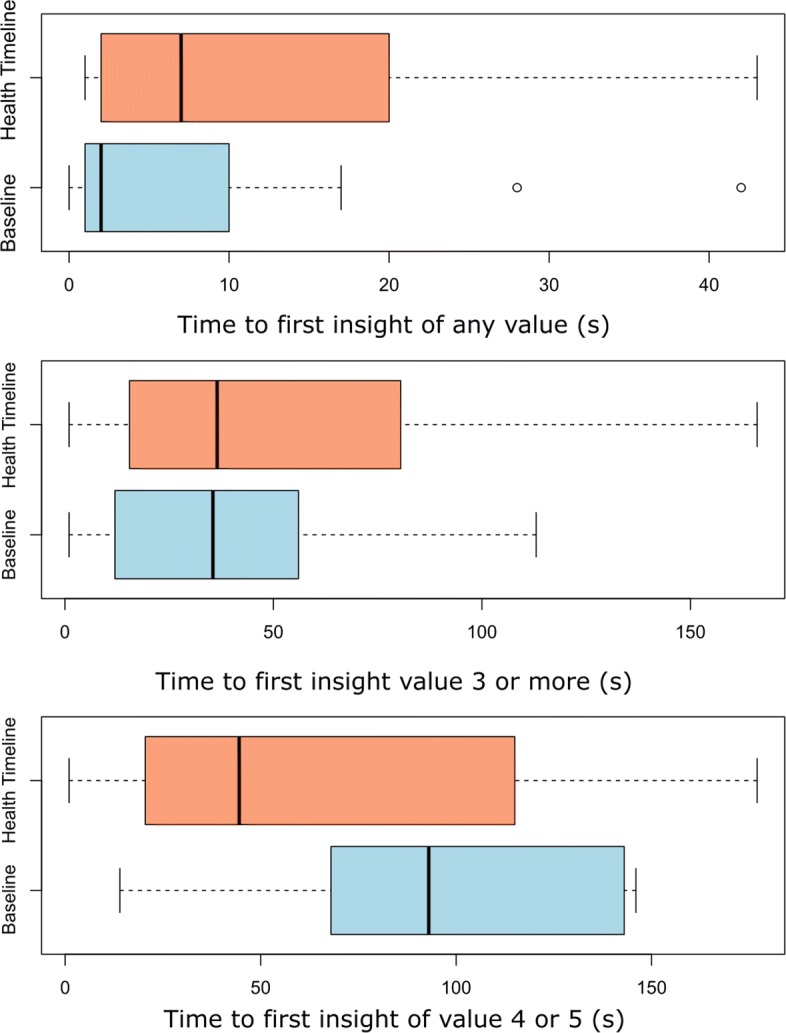
Time to first insight. The box plots represent the time to first insight of any value, value 3 or higher and value 4 or higher

The average time at which the participants formulated their first insight was analyzed. Figure 4 shows the distribution and Table 5 shows the calculated values with their statistical significance. For insights of any given value, the Timeline was slower (13.16 s) compared to the baseline (7 s; *p*<0.01). Insights with a value greater than one and two were also slower with the Timeline (22.24 s and 51.21 s) compared to the baseline (20.36 s; *p*=0.14 and 40.01 s; *p*<0.01). For insights with a value greater than 3, the Timeline was faster (68.44 s) compared to the baseline (92.83 s; *p*<0.01). Only the Timeline was able to generate insights of value greater than 4 with an average time of 63.50 s.

**Table 5:** Time to First Insight

Insight Value	Baseline	Health Timeline	Ratio	*p*-values
Any	*μ*=7	*μ*=13.16	1.88	<0.01
	*σ*=±10.02	*σ*=±14.24		
>1	*μ*=20.36	*μ*=22.24	1.09	0.17
	*σ*=±16.66	*σ*=±18.48		
>2	*μ*=40.01	*μ*=51.21	1.28	<0.01
	*σ*=±29.96	*σ*=±45.98		
>3	*μ*=92.83	*μ*=68.44	0.74	<0.01
	*σ*=±51.27	*σ*=±59.88		
>4	*μ*=0	*μ*=63.50	—	<0.01
	*σ*=0	*σ*=±60.06		

The table shows the mean and standard deviation of the time to first insight from any value to values 1 to 5. The ratio column shows the observations of the Health Timeline divided by the baseline. The *p*-values are obtained using Wilcoxon Signed-Rank Test

## Discussion

The insight-based methodology applied to this case study, provided an approach to quantify the degree to which a visualization tool assisted the process of understanding longitudinal clinical data. The design of the study enabled us to investigate the usefulness of a visualization in a real-world environment with medical doctors as participants of the study (domain experts and the intended audience for the visualization software).

The high number of low value insights from the baseline visualization could be partly explained by difficulty in putting together smaller units of information contained in each row of the tabular format. This, in turn made it difficult to derive deeper and more complex insights leading to a hypothesis. In contrast, the Health Timeline assisted participants in generating higher value insights at the cost of a longer time delay. Health Timeline could have encouraged the participants to take a more “deliberate” approach when making decisions about the data, which was in turn reflected in the insights derived during the assessments.

### Possible generalization of the results

It can be said that in the context of longitudinal clinical data, a time-based visualization of chronological events assists its audience better than textual information. In this context, the data visualization assisted the participants in gaining a greater understanding of the data (complete and accurate). In some cases, clinicians were able to understand the clinical history of the patients, formulate a diagnosis and suggest treatments.

This study shows the need to use a structured assessment methodology in the context of healthcare to determine the extent to which a visualization can assist clinicians to understand data. Without an objective assessment, it becomes subjective to state with confidence that one visualization is “useful” or “better”. Lam and colleagues [25], as well as Bertini and researchers [20] also emphasize the importance of structured assessment methods to evaluate data visualizations. This study also serves as a documented example of a contextualized assessment of visualizations in order to provide a use-case evaluation that is based on a real-life scenario. Even though the results of this study are encouraging, a larger study is warranted to examine objective outcomes and the impact that clinical data visualization may have in patient outcomes over longer periods of time.

### Limitations and considerations

To facilitate participation in our study, we applied the insight-based methodology with the “thinking aloud process”. All the audio recordings were transcribed for evaluation. The sample size and the clinical data used in this study required a large number of working hours for the transcription and evaluation of the insights. Increasing sample size or participants would improve representativeness and statistical reliability of the results but at the cost of a greater number of hours, which was outside our resources, unfortunately. Therefore, the generalizability of this study may be limited, and further studies would be desirable to confirm our findings.

Another limitation of the study was the time available to conduct the assessments. Even with the relatively brief time window of 30 min, we failed to recruit more subjects. This could probably be explained by the busy schedules of the psychiatrists.

The evaluation criteria, even though revised and peer-reviewed by domain experts, could also be subject to bias. A larger group of domain experts could provide more objective criteria. It is possible that experts that were not acquainted with the data might have provided better evaluation criteria, as these experts would have a fresh look at the clinical data without preconceived notions.

Given the results obtained in this study, we propose a further study to test if the visualization can assist a larger group of experts in providing higher quality of care to patients. Such a study would require a larger number of participants, a larger number of patients with the required ethical approval and a randomized controlled trial.

### Clinical data in a real healthcare context

The clinical data used in this study did not include diagnoses and notes taken by the practitioners during previous consultations. This was deliberate to allow the participants to come up with their own conclusions and assessments.

In a real life scenario, practitioners would have access to the diagnoses, notes and observations made by other clinicians. For the purpose of the study, we did not want to include this information to test the effectiveness of the visualization. The insights formulated in the assessments were our measurement to let us know the degree to which the participants were able to understand the clinical data without any previous information. We acknowledge that this is not an entirely realistic scenario however, for the purpose of the experiment, it allowed us to understand the usefulness of the visualization.

The clinical data was comparable to the reality of the healthcare context because it was extracted directly from real patients. In terms of complexity, we selected patients with a high degree of complexity of their treatment, in-patient events and diagnosis to base the study on complex real-life scenarios.

## Conclusion

This study detailed the assessment of a time-based EHR visualization software by applying the insight-based methodology. The results show that the Health Timeline data visualization tool supported the generation of valuable insights following the proposed criteria. Furthermore, the use of the assessment method demonstrates the feasibility of applying a consistent methodology to assess visualization techniques and tools in the clinical context. We propose that the insight-based methodology could be used in future studies as a methodological approach to assess the value of a clinical data visualizations.

## Additional file

Additional file 1 A comma-separated values data file containing the transcribed insights, with the annotations used for the study. The file is in a standard format and is machine-readable. The annotations of the file are detailed in the “Data analysis” section of this article. (CSV 24 kb)

## Data Availability

All data generated or analyzed during this study are included in this published article and its supplementary information files.

## Abbreviations

EHR: Electronic health record
MBS: Medicare Benefits Scheme
MHR: Medical health records
MWW: Mann–Whitney–Wilcoxon
NEHTA: National E-Health Transition Authority
PBS: Pharmaceutical Benefits Scheme
